# Taxonomic identification of the thermotolerant and fast-growing fungus *Lichtheimia ramosa* H71D and biochemical characterization of the thermophilic xylanase *Lr*XynA

**DOI:** 10.1186/s13568-017-0494-y

**Published:** 2017-11-02

**Authors:** María Teresa Alvarez-Zúñiga, Alejandro Santiago-Hernández, Johan Rodríguez-Mendoza, Jorge E. Campos, Patricia Pavón-Orozco, Sergio Trejo-Estrada, María Eugenia Hidalgo-Lara

**Affiliations:** 1 Departamento de Biotecnología y Bioingeniería, CINVESTAV-IPN, Av. Instituto Politécnico Nacional No. 2508, 07360 Mexico City, Mexico; 2 Laboratorio de Bioquímica Molecular UBIPRO., FES Iztacala, UNAMAv., de los Barrios No. 1, Los Reyes Iztacala, 54090 Tlanepantla, Estado de Mexico Mexico; 3 Facultad de Ciencias Químicas, Universidad Veracruzana, Av. Universidad Km, 7.5., 96538 Coatzacoalcos, Veracruz Mexico; 4 Centro de Investigación en Biotecnología Aplicada-IPN, Km 1.5 Carretera Estatal Tecuexcomac-Tepetitla, 90700 Tepetitla, Tlaxcala Mexico

**Keywords:** Internal transcribed spacer (ITS), Zygomycete fungus, *Lichtheimia ramosa*, Xylanase, Sugarcane hydrolysis

## Abstract

The zygomycete fungus *Lichtheimia ramosa* H71D, isolated from sugarcane bagasse compost, was identified by applying phylogenetic analysis based on the DNA sequence of the Internal Transcribed Spacer (ITS), and subsequent secondary structure analysis of ITS2. *L. ramosa* H71D was able to grow over a wide range of temperatures (25–45 °C), manifesting optimal growth at 37 °C. A 64 kDa xylanase (named *Lr*XynA) was purified from the culture supernatant of *L. ramosa* H71D grown on 2% carboxymethylcellulose (CMC), as the only carbon source. *Lr*XynA displayed optimal activity at pH 6 and temperature of 65 °C. The enzyme retained more than 50% of its maximal activity over a broad range of pH values (4.5–7.5). Enzyme half-life (t_½_) times at 55, 65 and 75 °C were 80, 25, and 8 min, respectively. *Lr*XynA showed higher affinity (*k*_*M*_ of 2.87 mg/mL) and catalytic efficiency (*k*_*cat*_/*k*_*M*_ of 0.651 mg s/mL) towards Beechwood xylan in comparison to other substrates such as Birchwood xylan, Oat-spelt xylan, CMC, Avicel and Solka floc. The predominant final products from *Lr*XynA-mediated hydrolysis of Beechwood xylan were xylobiose and xylotriose, suggesting that the enzyme is an endo-β-1,4 xylanase. Scanning electron microscopy (SEM) imaging of sugar cane bagasse (SCB) treated with *Lr*XynA, alone or in combination with commercial cellulases, showed a positive effect on the hydrolysis of SCB. To our knowledge, this is the first report focusing on the biochemical and functional characterization of an endo-β-1,4 xylanase from the thermotolerant and fast-growing fungus *Lichtheimia ramosa.*

## Introduction

Xylan is next in order to cellulose, in terms of the major structural components of plant cell walls, and is the second most abundant renewable polysaccharide in nature (Collins et al. 2002). Xylan is a complex, highly branched heteropolysaccharide and its structure varies between different plant species. The homopolymeric backbone chain of xylan consists of 1,4-linked β-d-xylopyranosyl units, that to a varied extent can be substituted with glucuronopyranosyl, 4-*O*-methyl-d-glucuronopyranosyl, α-l-arabinofuranosyl, acetyl, feruloyl or *p*-coumaroyl side-chain groups (Kulkarni et al. 1999). The complete hydrolysis of xylan requires the action of several enzymes, including endo-1,4-β-d-xylanase (EC3.2.1.8), which is crucial for xylan depolymerization (Polizeli et al. 2005). Xylanases, as glycoside hydrolase members, are able to catalyze the hydrolysis of the glycosidic linkage (β-1,4) of xylosides, leading to the formation of a sugar hemiacetal and the corresponding free aglycone (Hatanaka 2012). Xylanases and the microorganisms that produce them are interesting because they have extensive biotechnological applications. These enzymes are currently used in waste management in order to degrade xylan for the production of renewable fuels and chemicals. Likewise, they are used in food, agro-fiber, and paper and pulp industries, where xylanases help to reduce environmental impact (Collins et al. 2002). Oligosaccharides produced from the action of xylanases are further used as functional food additives or alternative sweeteners with beneficial properties (Pellerin et al. 1991). In terms of biotechnological application, thermostable enzymes have several generic advantages as the high specific activity, that is often associated with this kind of enzymes, reduces the required amount of enzyme and prolongs hydrolysis time due to greater stability than that exhibited by mesophilic enzymes (Viikari et al. 2007).

Many reports exist on thermophilic and mesophilic microorganisms signifying that bacteria and fungi are major producers of thermostable xylanases (Sunna and Antranikian 1997; Collins et al. 2002). In terms of fungi, most xylanases characterized to date are derived from Ascomycetes and Basidiomycetes; in particular, mesophilic fungi belonging to the genera *Aspergillus* and *Trichoderma* are preeminent in xylanase production (Polizeli et al. 2005). Production of thermostable xylanases has been reported for several filamentous fungi including *Laetiporus sulphureus* (Lee et al. 2009), *Talaromyces thermophiles* (Maalej et al. 2009), *Thermomyces lanuginosus* (Singh et al. 2003), *Nonomuraea flexuosa*, *Thermoascus aurantiacus* (Zhang et al. 2011), and the Zygomycete fungus *Rhizomucor miehei* (Fawzi 2011).

Zygomycetes are able to grow on a wide variety of carbon sources at different temperatures, oxygenation rates, and pH values (Ferreira et al. 2013). Recently, Zygomycetes are receiving increased attention in the biotechnological context for the production of a wide range of metabolic products e.g., organic acids, enzymes, and biofuels such as bioethanol and biodiesel (Ferreira et al. 2013). The genus *Lichtheimia* (syn. Mycocladus, Absidia) belongs to the Zygomycete class and includes saprotrophic microorganisms that can be isolated from decomposing soil and plant material (Alastruey-Izquierdo et al. 2010). Members of this genus are considered to constitute thermotolerant fungi, as they can grow at a wide range of temperatures, from 20 to 53 °C, with 37 °C presenting the best temperature for growth, where it occurs most rapidly (Voigt et al. 1999; André et al. 2014). This rapid growth rate of filamentous fungi belonging to the Zygomycete genus *Lichtheimia* makes them pertinent to the study of enzymes involved in the breakdown of plant material and offers possible advantages for a biotechnological application.

There are few studies on carbohydrate-active enzymes in the Zygomycetes fungi belonging to the *Lichtheimia* genus. *Lichtheimia blakesleeana* was described as a producer of phytase and xylanase (Neves et al. 2011). Additionally, *Lichtheimia ramosa* has been reported as a producer of xylanase, carboxymethylcellulase (CMCase), an ample producer of β-glucosidase in wheat bran-based medium (Gonçalves et al. 2013), and also a producer of amylases, β-glucosidases, CMCase, and xylanases, via solid state bioprocess, utilizing fruit waste from the Brazilian savannah (de Silva et al. 2013). Nevertheless, to our knowledge, there are no studies on the biochemical and catalytical properties of xylanases from a filamentous fungus belonging to the Zygomycete genus *Lichtheimia*.

Taxonomy of *Mucorales* has traditionally been based on microscopic morphology and mating experiments; however, molecular phylogeny has revealed that diversity within and between species is much greater than anticipated, also leading to a proliferation of the number of taxa recognized (Walther et al. 2013). The internal transcribed spacer region (ITS) consists of three parts: ITS1, ITS2 and the highly conserved 5.8S rDNA exon located between them. ITS2 usually has a conserved secondary structure with four helices which appear to be essential for successful excision of ITS2 from the precursor rRNA (Caisová et al. 2011). The ITS2 has been viewed as a possible useful marker for taxonomic classification, at a wide range of levels (Coleman 2003), because of its high divergence in sequence and assumed conservation in structure (Schultz et al. 2005). Additionally, it has been suggested modeling this cloverleaf-like structure as a novel tool for phylogenetics (Wolf et al. 2005). Furthermore, the ITS2 has been proposed as a candidate for the DNA fungi barcodes because it possesses a number of valuable characteristics (Yao et al. 2010). In *Mucorales*, the ITS region turned out to be an appropriate barcoding marker (Walther et al. 2013).

Hence, the aim of this work was to use phylogenetic analysis to identify the H71D strain, isolated from sugarcane bagasse compost, and undertake the purification and biochemical characterization of a secreted xylanase from this thermotolerant and fast-growing fungus.

## Materials and methods

### Microorganisms and growth conditions

The strain H71D was isolated from composting soils and kindly donated by Dr. Sergio Trejo-Estrada research group (CIBA-IPN, Tlaxcala. México).

For spore production, the fungus was grown on potato dextrose agar (PDA) medium plates and incubated at 37 °C for 72 h. Spore collection was performed and a suspension thereof, which was read at λ = 650 nm and adjusted to achieve an Absorbance of 0.5, which is equivalent to 5 × 10^6^ spores/mL (Tien and Kirk 1988).

For genomic DNA preparation and xylanase production, the strain H71D was grown in the liquid culture media described by Mandels and Sternburg (1967), with some modifications as follows. The culture basal medium contained in g/L: yeast extract 1; (NH_4_)_2_SO_4_ 1.4; KH_2_PO_4_ 2.0; Urea 0.3; CaCl_2_ 0.3; MgSO_4_ 7H_2_O 0.3; and 1 mL/L of trace element solution containing (g/L): FeSO_4_ 7H_2_O 0.05; MnSO_4_ H_2_O 0.016; ZnSO_4_ 7H_2_O 0.014; CoCl_2_ 0.02. The culture basal medium was supplemented with 2% (w/v) CMC as the only carbon source, unless otherwise stated. The medium was sterilized for 15 min at 121 °C and 15 psi. Flasks of 250 mL containing 50 mL of medium were inoculated with 500 µL of spore solution of the desired concentration, and flasks were incubated at 37 °C for 9–12 days on an orbital shaker at 160 rpm.

### Identification of the H71D strain

The identification of the strain H71D was made using morphological characteristics and the DNA sequence of the ribosomal DNA ITS2 region as a molecular marker. Morphological characterization followed the key published elsewhere (Hoffmann 2010). For molecular analysis, the genomic DNA was extracted from mycelia of H71D strain grown in liquid medium after 3 days of incubation at 37 °C and orbital agitation at 160 rpm, as described above. The mycelium was obtained by centrifugation (7000 rpm at 4 °C for 20 min); then, it was ground with liquid nitrogen, and this material was used for genomic DNA extraction, by using the DNeasy Blood & Tissue kit (QIAGEN, Valencia, CA). The ITS2 region was amplified from genomic DNA by PCR using the HotStar HiFidelity Polymerase Kit (Qiagen, Valencia, CA), and the barcoding primer pair ITS4 and ITS5 previously reported (White et al. 1990). The DNA sequence of the ITS2 region from H71D was compared with those ITS2 sequences from strains deposited at NCBI-GenBank, by using BLASTn available at the NCBI server (https://www.ncbi.nlm.nih.gov/). The phylogenetic analysis was carried out using ITS2 sequences from *Lichtheimia* species (Table 1), employing as outgroup the ITS2 sequence from *Dichotomocladium elegans* CBS 695.76 (GenBank Accession: HM999950), which has previously been used for the same purpose (O’Donnell et al. 2001). The multiple alignments were conducted by using the Clustal X program (version 2.0) (Larkin et al. 2007), and the FindModel program (https://www.hiv.lanl.gov/content/sequence/findmodel/findmodel.html) was used to select the model that best describes the data to generate a better tree. The phylogenetic analysis was conducted by the method of maximum likelihood using PhyML with 100 bootstrap replicates. The secondary structure of the ITS2 from the strain H71D was predicted by using the RNAfold WebServer (http://rna.tbi.univie.ac.at/cgi-bin/RNAfold.cgi), with the following setup: (i) minimum free energy (MFE), (ii) partition function and avoid isolated base pairs for fold algorithms, (iii) with no dangling end energies, and (iv) RNA parameters: Turner model (Mathews et al. 2004) at 37 °C. For comparison purpose, the ITS2 sequence from *L. ramosa* GQ342874 strain was selected and analyzed under the same conditions. Hence, the H71D strain was identified as *L. ramosa* H71D strain and deposited on “Colección de Cultivos Microbianos” (CDBB CINVESTAV-IPN, México) with access number CDBB–H–1939. The ITS2 DNA sequence from *L. ramosa* H71D strain was deposited in the GenBank database with Access Number KY311837.

**Table 1:** GenBank accession numbers of the DNA sequences from members of the genus *Lichteimia* used in this study

Strain	Specie	ITS GenBank Access Number
H71D	*L. ramosa*	KY311837
CBS 100.17	*L. corymbifera*	GQ342885
CBS 100.31	*L. corymbifera*	GQ342879
CBS 100.51	*L. corymbifera*	GQ342886
CBS 429.75	*L. corymbifera*	GQ342878
CBS 519.71	*L. corymbifera*	GQ342889
CBS 109940	*L. corymbifera*	GQ342881
CBS 100.28	*L. hyalospora*	GQ342896
CBS 100.36	*L. hyalospora*	GQ342898
CBS 102.36	*L. hyalospora*	GQ342895
CBS 173.67	*L. hyalospora*	GQ342893
CBS 518.71	*L. hyalospora*	GQ342894
CBS 291.66	*L. ornata*	GQ342891
CBS 958.68	*L. ornata*	GQ342890
CNM-CM 4978	*L. ornata*	GQ342892
AS 3.4808	*L. ramosa*	GQ342867
CBS100.24	*L. ramosa*	GQ342876
CBS100.49	*L. ramosa*	GQ342858
CBS 223.78	*L. ramosa*	GQ342877
CBS 271.65	*L. ramosa*	GQ342875
CBS 582.65	*L. ramosa*	GQ342874
CBS 649.78	*L. ramosa*	GQ342849
CBS 124197	*L. ramosa*	GQ342870
CBS 124198	*L. ramosa*	GQ342848
CNM-CM 3148	*L. ramosa*	GQ342872
CNM-CM 3590	*L. ramosa*	GQ342869
CNM-CM 4261	*L. ramosa*	GQ342854
CNM-CM 4337	*L. ramosa*	GQ342852
CNM-CM 4427	*L. ramosa*	GQ342853
CNM-CM 4537	*L. ramosa*	GQ342873
CNM-CM 4849	*L. ramosa*	GQ342855
CNM-CM 5111	*L. ramosa*	GQ342871
CNM-CM 5171	*L. ramosa*	GQ342864
CBS 420.70	*L. sphaerocystis*	GQ342900
CBS 647.78	*L. sphaerocystis*	GQ342899
CBS 648.78	*L. sphaerocystis*	GQ342901
CBS695.76	*D. elegans*	HM99995

### Determination of optimum growth temperature

To determine the optimum growth temperature, *L. ramosa* H71D strain was analyzed based on its radial growth (cm) on Petri dishes with PDA at different temperatures (25, 30, 35, 37, 40 and 45 °C) during 6 days. PDA plates were inoculated with small circles taken from the edge of a 2 days old colony. The plates were incubated at the different temperatures indicated above, and the diameter was measured every 8 h for 48 h. The growth rate, measured in centimeters per hour, was calculated for each plate and temperature.

### Enzyme and protein assays

Xylanase and cellulase activities were determined by measuring the amount of reducing sugars released, quantified by the DNS method at 540 nm (Miller 1959), using xylose or glucose as a standard. For xylanolytic activity, the assay mixture contained 25 µL of enzyme preparation, 975 µL of 0.2% Beechwood xylan (Sigma-Aldrich, St. Louis, MO, USA) in 50 mM citrate buffer, pH 6; subsequently, the mixture was stirred and incubated at 65 °C for 5 min. For cellulolytic activity, the assay mixture contained 100 µL of enzyme preparation, 900 µL of 0.3% CMC (Sigma-Aldrich, St. Louis, MO, USA) in 50 mM acetate buffer, pH 5.6; then, the mixture was stirred and incubated at 50 °C for 10 min. Enzyme activity was expressed as U/mL, where U corresponds to the µmoles of xylose/glucose released per minute, under assay conditions. All tests were carried out in triplicate and error bars represent the standard deviation. Protein concentration was measured using Bradford reagent (Sigma-Aldrich, St. Louis, MO, USA), and bovine serum albumin (Life Technologies, Grand Island, NY, USA) as the standard.

### Xylanase and cellulase production

For enzyme production, the H71D strain was grown in the liquid culture media as described by Mandels and Sternberg (1967), with some modifications as described above. Culture basal medium was supplemented with 2% (w/v) Beechwood xylan or 2% (w/v) CMC. The culture medium was sterilized for 15 min at 121 °C and 15 psi. Flasks of 2.8 L with 500 mL of medium were inoculated with 5 mL of spore solution at the desired concentration. Then, cultures were incubated at 37 °C for 9 days and orbital agitation at 160 rpm. Every 12 or 24 h, aliquot samples of 5 mL were taken from each flask. The pellet was obtained by centrifugation at 7000 rpm at 4 °C for 20 min, and was used to determine fungal biomass by the dry weight method; whereas the culture supernatant was used for extracellular enzyme (xylanase and cellulase) assays. The results presented are expressed as the mean ± standard deviation of three replicates.

### Enzyme purification

The culture supernatant (800 mL) was treated with ammonium sulfate (70% saturation). The precipitate was collected by centrifugation (8500 rpm, 4 °C for 15 min), then the pellet was resuspended and dialyzed against buffer A (50 mM Tris- HCl buffer pH 8, 0.1 mM PMSF, and 5% (v/v) glycerol). After dialysis, the protein preparation was loaded onto anion exchange UNOsphere Q (Bio-Rad), and cation exchange UNOsphere S (Bio-Rad) columns (column volume, 15 mL). Absorbed proteins were eluted from the column with a linear gradient of KCl (0.025–1 M) in buffer A, at a constant flow rate of 2 mL/min, and 2 mL fractions were collected. Fractions with xylanase activity were pooled and analyzed by 10% SDS-PAGE.

### Electrophoretic analysis

SDS–Polyacrylamide gel electrophoresis (SDS–PAGE) was performed using a polyacrylamide gel 10% according to the method described by Laemmli (1970). The gel was stained with Coomassie Brilliant Blue R-250 (Bio-Rad). Molecular weight (MW) was estimated by linear regression with reference to a broad range molecular weight protein standard (Bio-Rad).

### Zymogram analysis

The zymogram of xylanolytic activity was performed according to the methodology described by Royer and Nakas (1990), with some modifications as follows. Briefly, protein samples were separated on 10% polyacrylamide gels co-polymerized with 1% Remazol Brilliant Blue linked to xylan (RBB-X), under denaturing conditions. Protein samples were resuspended in SDS sample buffer with 5% (v/v) 2-β-mercaptoethanol, then samples were boiled in a water bath for 5 min. After electrophoresis, the gel was rinsed with distilled water and incubated in 50 mM citrate buffer, pH 6 at 50 °C for 2.5 h.

### Carbohydrate content

The amount of carbohydrates was determined by the Anthrone method (Leyva et al. 2008). A 0.2% of cold Anthrone solution was prepared in sulfuric acid. One milliliter of solution was slowly mixed with 500 µL of a sample preparation. This mixture was incubated at room temperature for 5 min, boiled for 10 min in a water bath, and then, tubes were placed on ice for 5 min. The samples were read at 640 nm, and the percentage of glycosylation was calculated according to the total amount of protein present in the sample. The standard curve was made with mannose.

### Biochemical properties

#### Optimal pH and pH stability

The effect of pH on the xylanolytic activity of *Lr*XynA was determined by varying the pH of the reaction mixtures using 50 mM citrate–phosphate buffer (pH 3–7), and 50 mM phosphate buffer (pH 6 to 8). Reaction mixtures were incubated at 50 °C for 10 min. For pH stability assay, the enzyme was preincubated in the above mentioned buffers, without substrate, at 50 °C for 3 h. Subsequently, the remaining xylanolytic activity was measured under standard conditions (65 °C and pH 6.0 for 5 min), and compared to the activity displayed by the untreated enzyme.

#### Effect of temperature on *Lr*XynA activity and stability

The effect of temperature on the enzymatic activity of *Lr*XynA was estimated by conducting the activity assay at different temperatures ranging from 30 to 80 °C in 50 mM citrate–phosphate buffer, pH 6. Reaction mixtures were incubated for 5 min under standard conditions. The thermostability of the enzyme was investigated after preincubation at 55, 65 and 75 °C without substrate. Residual enzyme activities at specific time points were determined under standard conditions, (65 °C, pH 6.0, for 5 min). To determine half-life (*t*½) of the enzyme, aliquot samples were withdrawn at different time intervals and residual enzymatic activity was measured under standard conditions.

#### Substrate specificity of *Lr*XynA and kinetic parameters

The xylanolytic activity of *Lr*XynA was determined under optimal assay conditions using 1% (w/v): Beechwood xylan, Birchwood xylan, Oat-spelt xylan, CMC, Avicel or Solka floc as the substrate. The kinetic parameters *k*_*M*_ and *V*_*max*_ of *Lr*XynA were determined under optimal conditions for enzyme activity using Beechwood xylan as substrate, at a concentration ranging from 0.05 to 1%. The kinetic parameters *k*_*M*_ and *V*_*max*_ were determined and calculated from the Nonlinear least squares regression applied to the Michaelis and Menten (http://statpages.org/nonlin.html).

#### Effect of metal ions and EDTA

To study the effect of various metal ions (Ca^2+^, Cu^2+^, Fe^2+^, Hg^2+^, Li, Mg^2+^, Mn^2+^, Na^+^, Ni^2+^ and Zn^2+^), and the chelating agent EDTA on the activity of *Lr*XynA, the enzyme was independently incubated with metal ions or EDTA, at final concentrations of 1 and 5 mM under optimal assay conditions (65 °C, pH 6 for 5 min). The activity was expressed as the percentage of the activity observed in the absence of any compound.

#### Analysis of *Lr*XynA hydrolysis products

Thin-layer chromatography (TLC) of *Lr*XynA hydrolysis product was carried out as follows. Purified enzyme (50 µL, 3 U/mL) was mixed with 50 µL of 1% (w/v) Beechwood xylan in 50 mM citrate buffer, pH 6. The reaction proceeded at 40 °C, and aliquot samples were taken after 0, 12, 24, 36, 48 h of incubation. TLC was carried out at room temperature using as mobile phase a mix of butanol:ethanol:water 5:3:2 (v/v). The plate was air dried, sprayed with H_2_SO_4_ 15% and bake at 100 °C for 2 h after color development.

#### Enzymatic hydrolysis of sugar cane bagasse (SCB)

SCB used in this work was previously characterized by Pavón-Orozco et al. (2012). The hydrolysis experiments were carried out to a final concentration of 15 mg/mL of SCB in a 50 mM citrate buffer pH 5, at 37 °C for 88 h and orbital shaking at 120 rpm. Enzymes used were, *Lr*XynA (X) from the *L. ramosa* H71D strain (this work), a commercial cellulase from *Aspergillus niger* (A) (Sigma-Aldrich, USA) and a commercial cellulase from *Trichoderma viridae* (T) (Calbiochem, USA), kindly donated by Dr. Plinio Guzmán Villate (CINVESTAV-Irapuato). In addition, reaction mixtures with different molar ratios from 0 to 100% of X combined with A or T were used. In all assays, the final molar concentration of the enzymes was kept constant at 34 mM. All preparations were supplemented with 0.01 mM β-glucosidase (Sigma-Aldrich, USA), to prevent potential inhibition by product, and with 0.02% sodium azide, to avoid contamination during kinetics. Aliquot samples (400 µL) were taken every 8 h during 88 h, time points were analyzed for reducing sugars (glucose as standard) by the DNS method (Miller 1959). The hydrolysis reaction was stopped by boiling the samples 5 min, followed by centrifugation at 10,000 rpm for 5 min. Hydrolysis experiments were performed in triplicate. A total amount of 15 mg/mL of SCB in 50 mM citrate buffer, pH 5.0, 0.01 mM β-glucosidase and sodium azide 0.02%, was used as a blank. For control one, the blank was supplemented with the specified proportion of X. For control two, the blank was supplemented with the specified proportion of A or T. Therefore, a blank and two controls were used for each reaction mixture and data obtained were used to calculate the amount of reducing sugars released from each reaction.

#### Scanning electron microscopy (SEM) analysis

The effect of xylanase/cellulase activity on the surface of SCB, before and after enzymatic treatments of SCB with xylanase (X) from the *L. ramosa* H71D strain (this work), and a commercial cellulase from *Aspergillus niger* (A) or from *Trichoderma viridae* (T), were analyzed by SEM imaging. Sample preparations for SEM were dried in a muffle at 50 °C, and then placed directly on a graphite layer, coated with gold and finally observed at 10 kV with an amplification of ×50 and ×3,000 on a JEOL (JSM 6510 LB) at the Electronic Microscopy Laboratory (CGSE, CINVESTAV-IPN, México).

## Results

### Identification of H71D strain

The H71D strain was identified based on morphological characteristics and molecular markers. Morphological characteristics of H71D strain were comparable to those described for the type species of *L. ramosa* (Hoffmann 2010), e.g., sporangia were light gray colored, subsporangial septum was absent, and sporangiospores were ellipsoidal (data not shown).

**Fig. 1:**
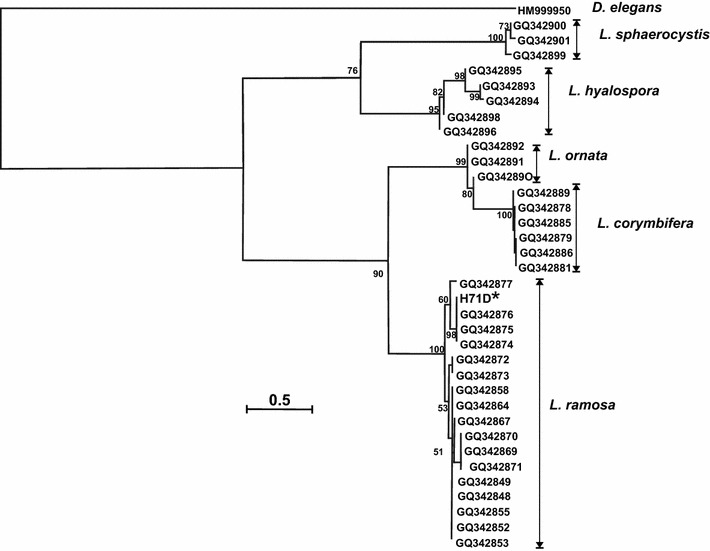
Phylogenetic tree inferred from ITS DNA sequences from *Lichtheimia* species. The tree was inferred under PhyML algorithm using an HKY85 model. The numbers on the branches correspond to the robustness (bootstrap values) obtained from 100 replicates

Taxonomic identification was carried out based on the DNA sequence of the ITS2, as a molecular marker. Thirty-six ITS2 DNA sequences from members of the genus *Lichtheimia* were selected from the GenBank for phylogenetic analysis and listed in Table 1. The phylogenetic tree was created by the method of maximum likelihood using the PhyML (HYK85 model) with 100 bootstrap replicates. Findings here indicate that the H71D strain belongs to *L. ramosa* clade, which was further corroborated by a high bootstrap value (Fig. 1). This molecular analysis also revealed that the ITS2 DNA sequence from H71D strain is very similar (98% of identity) to those sequences from *L. ramosa* GQ342876, GQ342875, and GQ342874.

**Fig. 2:**
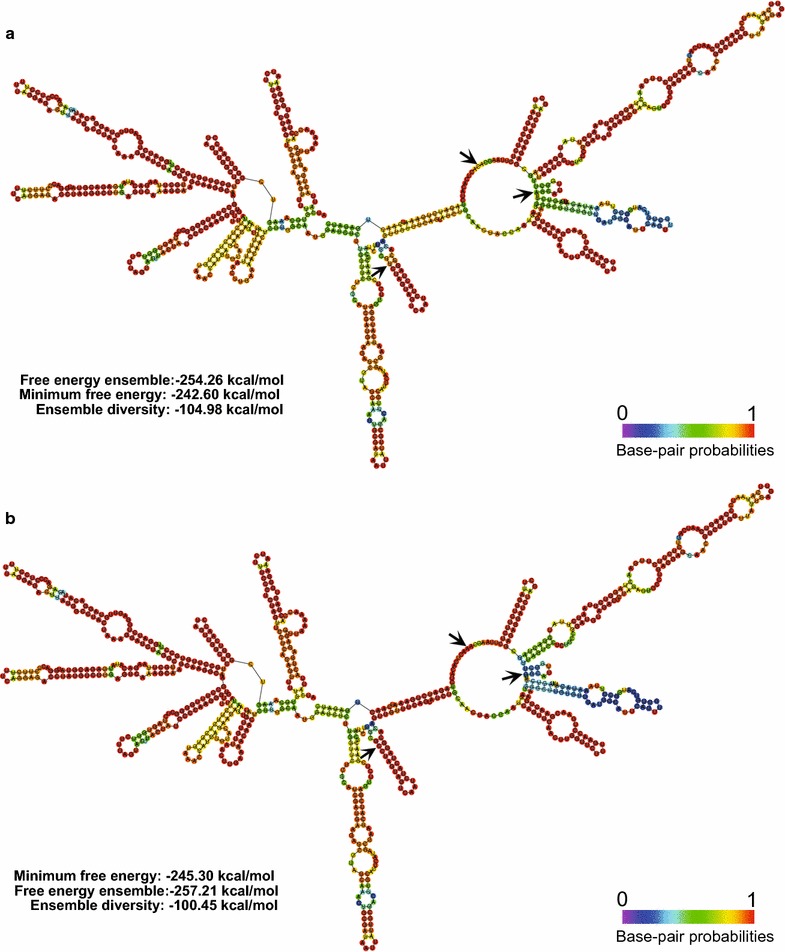
ITS2 secondary structures of strains **a** H71D and **b** GQ342874, determined with the RNA model Turner, 2004 (RNAfold Web Server); the most significant differences in the sequences that determined the secondary structure are indicated with black arrows

To confirm the identity of *L. ramosa* H71D, the secondary structure of the ITS2 region from strain H71D was compared to that from *L. ramosa* GQ342874, as both ITS2 regions are very similar (99% identity, 100% cover), and analyzed by RNAfold WebServer using the RNA Turner model, (Mathews et al. 2004) at 37 °C. Notably, the ITS2 DNA sequences from *L. ramosa* H71D and *L. ramosa* GQ342874 differ in only three nucleotides, corresponding to changes U→C, A→G and C→A, indicated by arrows (Fig. 2). These nucleotide changes did not result in different specific structures; however, the secondary structures showed differences in the MFE values of − 242.60 and − 245.30 kcal/mol for *L. ramosa* H71D and *L. ramosa* GQ342874, respectively.

### Determination of optimum growth temperature

**Fig. 3:**
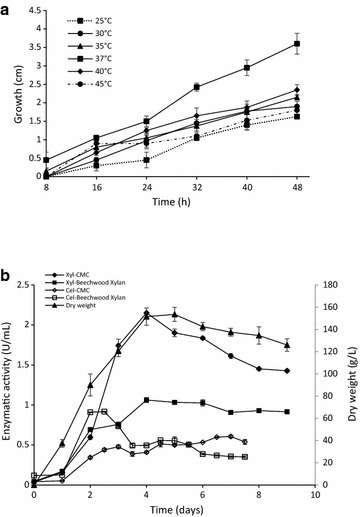
Xylanase and cellulose activities during growth of *L. ramosa* H71D. **a** Radial growth kinetic of *L. ramosa* H71D at different growth temperatures on PDA agar, the diameter was measured every 8 h for 48 h. **b** Growth and enzyme activities (xylanase and cellulose) of *L. ramosa* H71D at 37 °C. Dry weight in Mandels and Sternberg culture medium (Black up-pointing triangle). Xylanolytic activity produced on CMC (Black diamond suit) or Beechwood xylan (Black square), as a carbon source. Cellulolytic activity produced on CMC (Lozenge) or Beechwood xylan (Square), as a carbon source

To determine the optimum growth temperature of *L. ramosa* H71D, the fungus was cultured on Petri dishes with PDA medium at 25, 30, 35, 37, 40 and 45 °C for 6 days. The optimum growth temperature of *L. ramosa* H71D was 37 °C, with a radial growth of 3.6 cm after 48 h of incubation (Fig. 3a).

### Xylanase and cellulase production

**Fig. 4:**
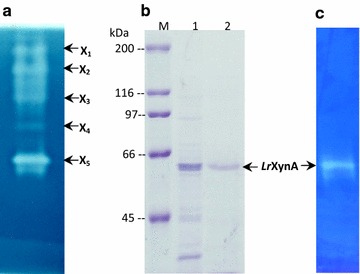
Protein and zymogram analysis of *Lr*XynA from *L. ramosa* H71D on 10% SDS-PAGE. **a** Zymogram analysis of crude extract from *L. ramosa* H71D, using 1% RBB-X as the substrate. **b** 10% SDS-PAGE analysis of purified *Lr*XynA. Lanes: M, molecular weight standard; 1, crude extract; 2, purified *Lr*XynA. **c** Zymogram analysis of purified *Lr*XynA, using 1% RBB-X as substrate

To evaluate the production of xylanase and cellulase activities, *L. ramosa* H71D was cultured at 37 °C in modified Mandels and Sternberg liquid media, using 2% (w/v) CMC or 2% (w/v) Beechwood xylan as carbon source. Xylanase and cellulase synthesis was induced with both carbon sources. Greatest xylanase activity was produced on CMC (2.1 U/mL) after 4 days of incubation, whereas the greatest cellulase activity was observed in the presence of Beechwood xylan (0.091 U/mL), after two and a half days of incubation (Fig. 3b). The xylanolytic activity onset occurred on the first day of culture, whereas highest activity was reached after 4 days of incubation; subsequently, xylanase activity remained constant for the next 4 days. Due to the xylanolytic activity was higher on CMC than on Beechwood xylan, the growth kinetic of *L. ramosa* H71D was investigated using CMC as the only carbon source (Fig. 3b). Zymogram analysis of the culture supernatant from *L. ramosa* H71D grown on CMC, after 4 days of incubation at 37 °C, revealed at least five bands with xylanolytic activity (Fig. 4a).

### Purification of *Lr*XynA

An extracellular xylanase was purified from the culture supernatant of *L. ramosa* H71D grown on CMC as the only carbon source. All purification steps are summarized in Table 2. The xylanase was purified 6.4-fold to homogeneity with a recovery yield of 38.5% and a specific activity of 126.43 U/mg of protein. The purified xylanase was separated on 10% SDS-PAGE, and its molecular weight was estimated to be 64 kDa (Fig. 4b), and named *Lr*XynA. Zymogram analysis of the purified *Lr*XynA, using 1% RBB-X as the substrate, showed a single clear band (Fig. 4c), thus confirming the xylanase activity of *Lr*XynA.

**Table 2:** Purification steps of xylanase *Lr*XynA from *L. ramosa* H71D

Sample	Total activity (U)	Total protein (mg)	Specific activity (U/mg)	Yield (%)	Purificación (n)
Crude extract	1878.01	95.12	19.75	100	1
70% ammonium sulfate precipitation	991.69	26.24	37.80	52.81	1.9
Ion exchange chromatography	723.12	5.52	131	38.5	6.6

### Carbohydrate content

Most xylanases produced by bacteria and fungi reported so far are glycosylated. The carbohydrate content of the enzyme was determined by the Antrone-sulfuric acid method, in order to reveal whether *Lr*XynA is a glycoprotein. However, no carbohydrate was detected.

### Biochemical properties

The purified xylanase *Lr*XynA from *L. ramosa* H71D was biochemically characterized and the results are described below.

### Effect of pH on *Lr*XynA activity and stability

**Fig. 5:**
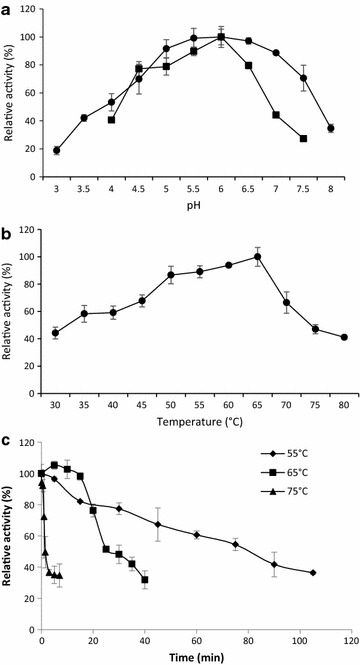
Effect of pH and temperature on *Lr*XynA activity and stability. **a** Effect of pH on xylanolytic activity (Black circle) and stability (Black square) of *Lr*XynA. *Lr*XynA was incubated in 50 mM citrate–phosphate (3–7) or phosphates (6–8) buffer and incubated at 50 °C for 10 min; for pH stability, *Lr*XynA was preincubated at 50 °C for 3 h in the same buffers. **b** Effect of temperature on the xylanolytic activity of *Lr*XynA. The enzyme was incubated in 0.2% (w/v) Beechwood xylan in 50 mM citrate–phosphate buffer, pH 6.0 at different temperatures (30–80 °C). **c** Thermostability of *Lr*XynA at 75 °C (Black up-pointing triangle), 65 °C (Black square) and 55 °C (Black diamond suit)

The influence of pH on the xylan hydrolysis of *Lr*XynA was determined at pH values ranging from 3 to 8 at 50 °C. *Lr*XynA showed maximum activity at pH 6 and exhibited about 50% of its maximal activity at different pH values ranging from 4 to 7.5 (Fig. 5a). The pH stability of *Lr*XynA at different pH values in the range from 3 to 8, after 3 h of incubation at 50 °C was evaluated. *Lr*XynA was stable at a broad range of pH (4.5–7), retaining more than 50% of its original activity (Fig. 5a).

### Effect of temperature on *Lr*XynA activity and stability

The effect of temperature on the xylan hydrolysis of *Lr*XynA was determined at different temperatures ranging from 30 to 80 °C, at pH 6. *Lr*XynA showed optimal activity at 65 °C, and displayed 50% of its maximal activity over a wide temperature range; from 35 to 70 °C (Fig. 5b). In order to evaluate enzyme thermostability, the purified *Lr*XynA was incubated at 55, 65 and 75 °C in 50 mM citrate–phosphate buffer, pH 6. The half-lifes of *Lr*XynA at 55 and 65 °C were 80 and 25 min, respectively (Fig. 5c).

### Substrate specificity of *Lr*XynA and kinetic parameters

The substrate specificity of *Lr*XynA was determined under optimal assay conditions, using 1% (w/v) Beechwood xylan, Birchwood xylan, Oat-spelt xylan, CMC, Avicel and Solka floc. *Lr*XynA showed high specificity for all of the xylans assayed, manifesting highest affinity to Beechwood xylan, whereas no activity was detected on CMC, Avicel and Solka floc. To determine the kinetic parameters *k*_*M*_ and *V*_*max*_, the initial reaction rates for *Lr*XynA were studied under optimal conditions for enzyme activity using Beechwood xylan as the substrate, at a concentration ranging from 0.05 to 1%. *Lr*XynA exhibited typical kinetics of Michaelis–Menten, with *k*_*M*_ and *V*_*max*_ values of 2.421 mg/mL and 6.325 U/mg, respectively (Table 3).

**Table 3:** Kinetic properties of *Lr*XynA

	*k*_*M*_ (mg/mL)	*V*_*max*_ (U/mg)	*kcat* (s^−1^)	Catalytic efficiency (mL/mg∙s)
Beechwood xylan	2.421	6.325	0.403	0.166
Birchwood xylan	5.155	8.726	0.553	0.107
Oat-spelt xylan	11.66	11.924	0.756	0.065
CMC	0	0	0	0
Solka floc	0	0	0	0
Avicel	0	0	0	0

### Effect of metal ions on enzyme activity

The effect of several metal ions and EDTA on the enzymatic activity of *Lr*XynA was determined at a final concentration of 1 and 5 mM each (Table 4). Xylanase activity of *Lr*XynA increased 170, 217 and 298% in the presence of the metal ions Ca^2+^, Mn^2+^ and Fe^2+^ (5 mM), respectively. The Mn^2+^ ion increased the activity of *Lr*XynA to 137 and 217% at a concentration of 1 and 5 mM, respectively; in contrast, the quelant agent EDTA decreased the enzymatic activity of *Lr*XynA by 3 and 16%, at concentrations of 1 and 5 mM, respectively. The activity of *Lr*XynA was almost completely inhibited by the Hg^2+^ ion at 1 and 5 mM (Table 4).

**Table 4:** Influence of metal ions and EDTA on the xylanolytic of *Lr*XynA

Metal ions and EDTA	Relative xylanase activity (%)	
	1 mM	5 mM
Control	100	100
Ca^2+^	95.2 ± 2.6	170.5 ± 9.7
Cu^2+^	96.7 ± 8.2	72.9 ± 2.4
Fe^2+^	157.7 ± 0.8	298.3 ± 4.2
Hg^2+^	7.5	7.9 ± 6.0
Li^+^	98.4 ± 6.2	121.8 ± 4.4
Mg^2+^	119.4 ± 0.5	123.1 ± 11
Mn^2+^	137.3 ± 4.6	217.7 ± 4.1
Na^+^	105.9 ± 5.15	119.6 ± 6.3
Ni^2+^	103.7 ± 7.9	122.5 ± 13
Zn^2+^	132.2 ± 2.5	143.1 ± 11.4
EDTA	97.3 ± 4.9	84.6 ± 9.9

### Analysis of *Lr*XynA hydrolysis products

**Fig. 6:**
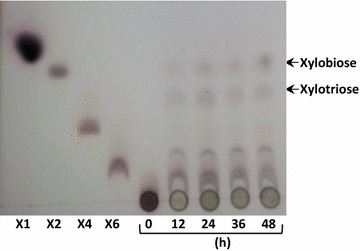
TLC analysis of Beechwood xylan by *Lr*XynA trough kinetic time of 0, 12, 24, 36 and 48 h. Standards: X1 (xylose), X2 (xylobiose), X4 (xylotetrahose) and X6 (xylohexosa)

The mode of action of *Lr*XynA towards Beechwood xylan was examined by analyzing the production of reducing-sugar at different times, and the hydrolysis products by silica gel thin-layer chromatography (TLC) (Fig. 6). The mobility of hydrolysis products after 48 h of incubation showed that the main products were xylotriose and xylobiose (Fig. 6).

### Enzymatic hydrolysis of sugarcane bagasse (SCB)

The xylanase *Lr*XynA from *L. ramosa* H71D (named as X) was functionally characterized by its ability to released reduced sugars from SCB alone, or in combination with the commercial cellulase from *Aspergillus niger* (named as A) or the commercial cellulase from *Trichoderma viridae* (named as T). The enzymatic hydrolysis of SCB (15 mg/mL) was evaluated by either X, A or T (100%), and with mixtures of X-A or X-T at different molar ratios (25:75, 50:50 and 75:25). In all cases, the reaction mixtures were supplemented with β-glucosidase to avoid inhibition by product, maintaining a final enzyme concentration at 34 mM. In control experiments with all substrates used, β-glucosidase alone did not produce any detectable reducing sugars. The tests were carried out for up to 88 h, however, 64 h was considered the final point because, after this time lapse, the release of reducing sugars ceases to be linear. After 64 h of hydrolysis, the xylanase X released 1.54 ± 0.07 µmol/mL, the cellulase A released 4.98 ± 0.28 µmol/mL and the cellulase T released 4.16 ± 0.99 µmol/mL of reducing sugars. The hydrolysis at different molar ratios (100, 25:75, 50:50 and 75:25) was evaluated for the mixtures of X-A or X-T. The maximum degradation of SCB was detected in the molar ratio of 25X: 75A/T at 64 h. The mix X-T released 4.77 ± 0.38 µmol/mL, whereas X-A released 5.66 ± 0.37 µmol/mL of reducing sugars after 64 h.

### Scanning electron microscopy (SEM) analysis

**Fig. 7:**
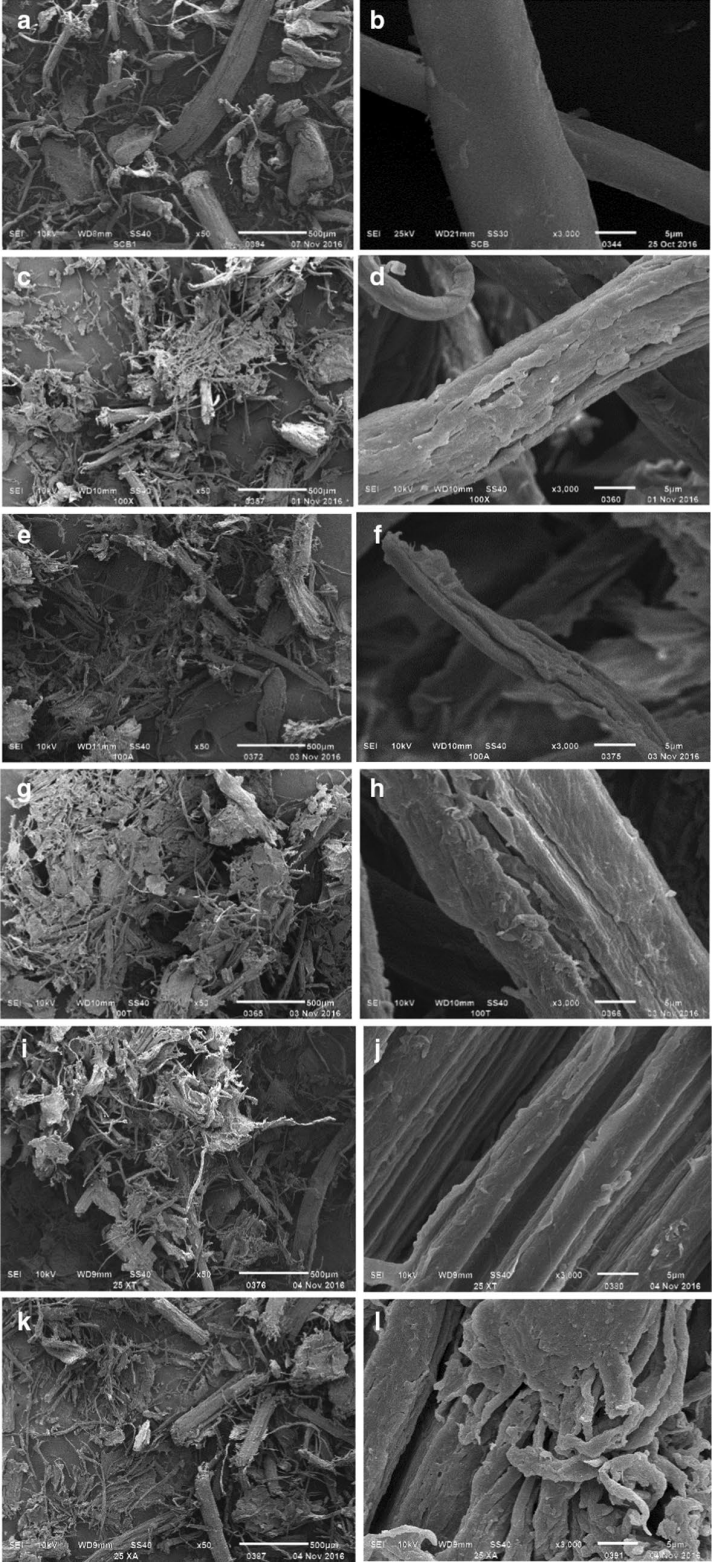
SEM analysis of untreated SCB samples (**a**, **b**) and with enzymatic treatment at 37 °C during 64 h of incubation. Using 100% of *Lr*XynA from *L. ramosa* H71D (**c**, **d**); 100% of cellulase from *A. niger* (**e**, **f**); 100% of the cellulase from *T. viridae* (**g**, **h**); a mixture (25:75) of *Lr*XynA/cellulase from *A. niger* (**i**, **j**), and a mixture (25:75) of *Lr*XynA/cellulase from *T. viridae* (**k**, **l**). On the left side the micrographs are shown with an amplification of ×50, and on the right side are shown with an amplification of ×3000

To evidence the impact of the purified xylanase *Lr*XynA (X) on the surface of SCB, as well as the effect of this enzyme in combination with commercial cellulases (A, T), SEM imaging of saccharified SCB was analyzed after 64 h of incubation at 37 °C (Fig. 7). First, to determine the effect of xylanase X on the surface of SCB, the reaction mix 100% X was assayed. Then, this methodology was used to evaluate a putative cooperative effect between xylanase X and a commercial cellulase A or T. For these experiments, reaction mixtures with different molar ratios (from 0 to 100%) were prepared. For all treatments involving a single enzyme, the biomass surface and fibrils became rough and disordered, possibly due to the removal of a polysaccharide (Fig. 7, panels C to H), compared to that observed for SCB without enzymatic treatment (blank), where the biomass surface appears integrated and the fibers look plane, smooth and continuous (Fig. 7, panels a, b). When *Lr*XynA was used in isolation, the fiber showed greater porosity and separation of microfibrils (Fig. 7, panel d). Interestingly, when the SCB was treated with a mix of xylanase/cellulase (25X:75 A or T), the surface was disrupted and looked scaly or cracked (Fig. 7, panels i–l).

## Discussion

Several lignocellulolytic enzymes produced by different microorganisms have been studied. In the context of fungi, Ascomycetes and Basidiomycetes have been widely studied; however, very few studies focus on Zygomycetes and their enzymes. This is why our study focused on the biochemical and functional characterization of one of the enzymes involved in the xylanolytic activity produced by the thermotolerant Zygomycete, *L. ramosa* H71D.

Firstly, we identified the H71D strain by its morphological characteristics and the molecular marker ITS2 as *L. ramosa* H71D. Phylogenetically, *Mucorales* constitute a very old group with considerable molecular distances between species. The weighted intraspecific ITS variability for Zygomycetes is 3.2% (Pawłowska et al. 2013); whereas for Ascomycetes vary by 1.96% (Nilsson et al. 2008). Furthermore, Walther et al. (2013) emphasize the fact that the intraspecific variability in *Mucorales* differs among species but can reach more than 5%, as occurs in *Mucor circinelloides* (5.3%) or *L. ramosa* (7.6%). It has been reported that the ITS2 secondary structure analysis can improve the phylogenetic resolution obtained from the primary sequence (Keller et al. 2008), and the combination and simultaneous analysis of sequence and structural ITS2 RNA data supplemented with indel coding binaries yielded robust phylogenetic hypotheses as measured by bootstrap values for ancestral haplotypes (Poczai et al. 2015). Moreover, Alastruey-Izquierdo et al. (2010) studied species boundaries in *Lichtheimia* using genealogical concordance phylogenetic species recognition and established that the ITS region is the marker of choice for molecular identification of species in *Lichtheimia* because of its high degree of variability and the possibility of direct sequencing in most cases. Therefore, due to the intraspecific ITS variability observed for *L. ramosa*, and with the aim to give more robustness to the identification of the H71D strain, the secondary structure of the ITS2 region from the H71D strain was analyzed and compared to that from *L. ramosa* GQ342874, because it is one of the sequences with which the H71D strain showed greater identity (99% identity, 100% cover). The ITS2 secondary structures obtained are the same; however, they differ in terms of MFE, − 242.60 kcal/mol (H71D strain) and − 245.30 kcal/mol (*L. ramosa* GQ342874), due to the three differences in the nucleotide sequence. Hence, phylogenetic analysis based on the ITS2 DNA sequence, and subsequent ITS2 secondary structure analysis allow us to identify the H71D strain as *L. ramosa* H71D, with a high degree of certainty.

*Lichtheimia ramosa* H71D was able to grow over a wide range of temperatures (25-45 °C), manifesting optimal growth at 37 °C. A 64 kDa xylanase (named *Lr*XynA) was purified from the culture supernatant of *L. ramosa* H71D grown on 2% carboxymethylcellulose (CMC), as the only carbon source.

The optimum growth temperature of *L. ramosa* H71D was determined as 37 °C and the colony reached 3.6 cm after 48 h of incubation. In agreement to our data, an optimum temperature for *L. ramosa* growth of 35 °C, based on its extensive radial growth (5 cm) after 40 h of incubation, was reported (Gonçalves et al. (2013). According to the optimum growth temperature of *L. ramosa* H71D (37 °C), this fungus is mesophilic in nature; however, *L. ramosa* H71D can be considered a thermotolerant fungus because it is able to grow over a wide range of temperature (25–45 °C). Findings here concur with previous reports, e.g., in a study on *Mucorales* it was observed that thermotolerant species had optimum growth temperatures above 37 °C, between 37 and 45 °C (Hoffmann et al. 2007); in particular, it was reported that *L. ramosa* grew at a temperature that ranged from 24 to 49 °C (Alastruey-Izquierdo et al. 2010).

The xylanolytic activity produced by *L. ramosa* H71D in the presence of Beechwood xylan or CMC was assessed, indicating that xylanase activity was greater when the fungus was cultured on CMC compared to than that observed for Beechwood xylan as carbon source; thus, indicating that CMC is more effective for the production of xylanase activity by *L. ramosa* H71D, under the culture conditions tested. Similarly, it was reported that cellulose, cellobiose, and even heterodisaccharide, composed of glucose and xylose, induce the production of xylanolytic enzymes in *Aspergillus terreus* (Hrmová et al. 1991). The fungus *T. reesei* also exhibits cellulolytic and xylanolytic activity in the presence of cellulose, xylan, or mixtures of plant polymers (Amore et al. 2013). Interestingly, and in agreement to findings here, when *Neurospora crassa* was cultured on Avicel as the sole carbon source, both cellulases and hemicellulases encoding genes were induced, and the expression levels of some hemicellulases genes were much higher than those observed when *N. crassa* was cultured on xylan (Sun et al. 2012; Amore et al. 2013). The production of xylanases by fungi grown on cellulose as the only carbon source has been reported in fungi as *Hypocrea jecorina* (Stricker et al. 2008) and *Trichoderma harzianum* (Hrmová et al. 1989). Xylanase production by *L. ramosa* H71D (2.1 U/mL) is comparable to that reported for *L. ramosa* (1.80 U/mL) via solid state bioprocess, utilizing waste from Brazilian savannah fruit (de Silva et al. 2013); and for *L. ramosa* (2.54 U/mL) grown in wheat bran-based medium (Gonçalves et al. 2013).

In this study, we have purified, characterized, and quantitatively evaluated the activity of a xylanase from *L. ramosa* H71D. Zymogram analysis of *Lr*XynA using 10% SDS-PAGE revealed a band with an estimated MW of approximately 64 kDa, with xylanolytic activity. Most xylanases produced by bacteria and fungi are proteins pertaining to a subunit with a wide molecular weight range of 8–145 kDa (Beg et al. 2001). *Lr*XynA is not a glycoprotein; however, it has been estimated that over half the proteins in nature are glycosylated (Apweiler et al. 1999). Reports indicate that in the case of xylanases, carbohydrate decoration on β-xylosidases contributes 10–30% of their molecular weight. Exceptionally, fungal β-xylosidases from *Humicola grisea* var. *thermoidea* and *Paecilomyces thermophila* are not glycosylated (Knob and Carmona 2010) and four xylanases (xyn10A, xyn10B, xyn11A, xy11B) from *Penicillium oxalicum* GZ-2 are not glycosylated (Liao et al. 2015).

We compared certain biochemical characteristics of *Lr*XynA with those of other fungal xylanases. The optimal pH assay showed that the enzyme had maximal activity at 6, a value which falls within the range (2–8) of optimal pH values for several fungal xylanases (Beg et al. 2001). Xylanases obtained from different microorganisms with optimal function at pH 6 have been reported, such as those from *P. oxalicum* GZ-2 (Liao et al. 2015), *Humicola insolens* Y1 (Shi et al. 2015), *Remersonia thermophila* CBS 540.69 (McPhillips et al. 2014). The pH stability data of *Lr*XynA (4.5–7) was similar to other isolated xylanases, e.g., the xylanase from *R. miehei* retained more than 90% of its activity at pH values of 5 and 6.5 after 60 min at 50 °C (Fawzi 2011); whereas, the xylanase from *Thielaviopsis basicola* exhibited alkaline stabilities ranging from pH 3–11 (Goluguri et al. 2012), and the xylanase from *Chaetomium* sp. retained more than 80% of its activity after 30 min at 50 °C, when tested at the pH range from 4.5 to 11 (Jiang et al. 2010). The optimal temperature for *Lr*XynA was 65 °C, xylanases from other microorganisms exhibited an optimal activity at 65 °C, for example, that from *Paenibacillus* sp. DG-22 (Lee and Lee 2014) and *Remersonia thermophila* CBS 540.69 (McPhillips et al. 2014). This value falls within the range of optimal temperature values of several fungal xylanases. For example, 40 °C is the optimal temperature for the xylanase from *Leucoagaricus gongylophorus* (Moreira et al. 2014). Recently, our research group reported an optimal temperature of 85 °C for the xylanase TtXynA from the thermophilic fungus *Thielavia terrestris* Co3Bag1, which at that moment was the highest optimal temperature for fungal xylanases (García-Huante et al. 2017). Nevertheless, there are reports of non-fungal xylanases with optimal temperatures higher than 85 °C, such as SSO1354 from *Sulfolobus solfataricus* at 95 °C (Maurelli et al. 2008) and XYNB from *Dictyoglomus thermophilum* at 100 °C (Li et al. 2013). Thermostability assays indicated that *Lr*XynA displays low thermostability at 75 °C; however, the enzyme exhibited half-lifes of 25 and 80 min at 65 and 55 °C, respectively. At 65 °C, the thermostability of *Lr*XynA (t_½_ = 25 min) is lower than that displayed by the xylanase TtXynA (t_½_ = 23 days) from *T. terrestris* Co3Bag1 (García-Huante et al. 2017) but higher than that reported for the xylanase XynAS9 (t_½_ = 16 min) from *Streptomyces* (Wang et al. 2011).

It has been reported that over 90% of Beechwood and Birchwood xylan are composed of xylose. In Beechwood xylan, xyloses are mainly linked by 2,4 and 1,4-linkages; in Birchwood xylan, xyloses are mainly linked by 1,4-linkages, whereas most of the Oat-spelt xylan contains xylose and arabinose with minor amounts of glucose and galactose (Liab et al. 2000). Hence, our results suggest that *Lr*XynA has higher affinity towards 1,4-linkages between xyloses, present in Beechwood and Birchwood xylans, but when the amount of xylose decreases, as in Oat-spelt xylan, its affinity also decreases. Other reported xylanases show higher affinity for Beechwood xylan than for Birchwood xylan, e.g., XynGR40 (*k*_*M*_ = 1.8 mg/mL) from the environmental DNA of goat rumen (Wang et al. 2011) and TtXynA (*k*_*M*_ = 0.41 mg/mL) from *T. terrestris* Co3Bag1 (García-Huante et al. 2017). However, there are reports of other xylanases that show greater affinity for Oat-spelt xylan than for Beechwood xylan (Liao et al. 2015). *Lr*XynA did not show activity on CMC, Solka floc, and Avicel, and reports describe some other xylanases such as xyn10A, xyn10B, xyn11A, xy11B) from *P. oxalicum* GZ-2 to be active towards polymeric xylans; although not on other substrates (Liao et al. 2015). The kinetic parameters of *Lr*XynA and the *k*_*M*_ and *V*_*max*_ values, determined for each of the xylan substrates used in this study, are similar to those obtained for other fungal xylanases. The *Lr*XynA *k*_*M*_ value of 2.42 mg/mL is similar to those reported for rXynSW3 xylanase from *Streptomyces* sp. SWU10 of *k*_*M*_ = 2.3 mg/mL (Sukhumsirichart et al. 2014) and xylanase XYN2 from *T. reesei* of *k*_*M*_ = 2.1 mg/mL (He et al. 2009). Likewise, xylanases with *k*_*M*_ values higher than that observed for *Lr*XynA have been reported, e.g., endo-1,4-beta xylanase B (*k*_*M*_ = 8.9 mg/mL) from *Aspergillus niger* BCC14405 (Krisana et al. 2005) and Xyn II (*k*_*M*_ = 5.56 mg/mL) from *Aspergillus usamii* (Zhou et al. 2008). The *V*_*max*_ value (6.325 U/mg) of *Lr*XynA is higher than the *V*_*max*_ value reported for rXynSW3 (0.35 U/mg) of *Streptomyces* sp. (Sukhumsirichart et al. 2014), but it is lower than those reported for other xylanases, e.g., xylanase (*V*_*max*_ = 1235 U/mg) of *Talaromyces thermophilus* (Maalej et al. 2009) and xylanase (*V*_*max*_ = 113.5 U/mg) of *R. miehei* (Fawzi 2011).

The general consensus opines that some metal ions and reagents significantly affect xylanase activities (Juturu and Wu 2012). Therefore, we evaluated the effect of metal ions and EDTA on the xylanolytic activity of *Lr*XynA. The Fe^2+^ ion 5 mM is presented as the major activator for increasing the activity of the xylanase *Lr*XynA from *L. ramosa* by 298%. However, it has been reported that Fe^2+^ 1 mM inhibits the activity of the XYN11A from *P. oxalicum* by 68% (Liao et al. 2014). The Mn^2+^ ion increased the activity of *Lr*XynA by 137 and 217% at a concentration of 1 and 5 mM, respectively. The activity of a xylanase from *T. lanuginosus* DSM 5826 was also stimulated by 137% (Lin et al. 1999), whereas a 40% decrease was observed for a xylanase from *Streptomyces rameus* (Li et al. 2010), in the presence of the metal ion Mn^2+^. The metal ion Hg^2+^ is known to be toxic to enzymes, as it binds to thiol groups present in the active sites of the enzyme, causing irreversible inactivation. This ion Hg^2+^ (1 and 5 mM) decreased the xylanolytic activity of *Lr*XynA by 93%. It also inhibits 3 out of 4 xylanases (xyn10A, xyn10B, xyn11B) from *P. oxalicum* (Liao et al. 2015). Other reports state that the Hg^2+^ ion did not completely inhibit xylanase activity e.g., the xylanase xyn11A (24%, at 10 mM) from *P. oxalicum* (Liao et al. 2015), and the xylanase TtXynA (55%, at 1 mM) from *T. terrestris* (García-Huante et al. 2017). We also assessed EDTA, a metal chelator that may decrease xylanase activity. This would suggest that the enzyme needs a metal as a cofactor (Knob and Carmona 2010). The chelating agent EDTA, at final concentrations of 1 and 5 mM, decreased the activity of *Lr*XynA by 3 and 16%, respectively. A decrease of 9% in the activity of a xylanase from *L. sulphureus* in the presence of EDTA 5 mM was reported by (Lee et al. 2009).

TLC analysis of final products from hydrolysis of Beechwood xylan by *Lr*XynA were mainly xylotriose and xylobiose; therefore, *Lr*XynA can be classified as an endo-xylanase without β-xylosidase activity, as xylose was not observed as a product even after 48 h of incubation. According to Knob and Carmona (2010) xylotriose is the smallest oligomer produced by most known xylanases. Nevertheless, other xylanases from fungi, such as *L. sulphureus* (Lee et al. 2009) and *P. oxalicum* (Liao et al. 2014) hydrolyze xylans to predominantly produce xylobiose and xylotriose.

The individual action of xylanase *Lr*XynA (X), a commercial cellulase from *Aspergillus niger* (A), and a commercial cellulase from *Trichoderma viridae* (T) in the hydrolysis of SCB, as well as the effect of *Lr*XynA in combination with a commercial cellulase (A or T), was quantified by the liberation of reducing sugars during the hydrolysis of SCB. Data obtained indicated that a positive effect in the hydrolysis of SCB occurred mainly in both of the mixtures studied; X-T and X-A. To reveal the impact of xylanase *Lr*XynA, alone and in combination with commercial cellulases A or T, on the surface of SCB, samples of this substrate before and after enzymatic treatments were analyzed by SEM imaging. The micrographs showed the damage carried out by each of the enzymes separately; nevertheless, the damage was more noticeable when the enzymes were mixed (X-T and X-A). In this case, when xylanase *Lr*XynA (X) was used in isolation, the fiber showed greater porosity and separation of microfibrils; a similar effect was observed with the xylanase QG-11-3 from *Streptomyces* sp. on eucalyptus kraft pulp (Beg et al. 2000). Furthermore, when SCB was treated with the cellulases A or T, the layers of fiber appeared to break, exposing the inner channels of the fiber. Our findings concur with those that describe the action of CELULASE CE 2 from *Trichoderma longbrachiatum* (Proenzimas, Cali, Colombia) on SCB (Quintero and Cardona 2009). Hence, a clear positive effect on the hydrolysis of SCB was observed when *Lr*XynA was combined with any of the commercial cellulase preparations. This is probably due to the removal of hemicellulose by the action of *Lr*XynA, which leads to an improved enzymatic hydrolysis of cellulose fibers by the action of the commercial cellulases. A synergic effect that occurs between two individual non-complexed pure enzymes of two aerobes: CflXyn11A xylanase from *Cellulomomas flavigena* and TrCel7B cellulase from *Trichoderma reesei*, during the hydrolysis of sugarcane bagasse has been previously reported by our research group (Pavón-Orozco et al. 2012). Overall, data obtained in this work suggest that *Lr*XynA may represent an efficacious candidate for the degradation of plant cell biomass.

On the basis of morphological characteristics, the H71D strain was identified as *L. ramosa* (sporangia were light gray colored, subsporangial septum was absent, and sporangiospores were ellipsoidal). Phylogenetic analysis was based on the molecular marker ITS and its secondary structure. The study of new strains, especially those exhibiting fast-growing, as well as the biochemical characterization of its enzymes is important not only from an evolutionary point of view but also from an economic one, as demand has increased in the agricultural context with the production of biofuels from agricultural wastes.

There are few reports on glycosyl hydrolases produced by members of the *Lichtheimia* genus. To our knowledge, this study represents the first report on biochemical and functional characterization of a xylanase from a filamentous fungus belonging to the Zygomycete genus *Lichtheimia.* The xylanase *Lr*XynA from *L. ramosa* H71D is considered heat-tolerant and heat stable; biochemical properties that make it appropriate for application in different biotechnological processes such as the biofuel industry, the manufacture of bread and animal feed and in the treatment of lignocellulosic residues. The bioconversion of hemicellulose to value-added products using xylanases thus offers many promising applications.

## Abbreviations

ITS: internal transcribed spacer
*Lr*XynA: xylanase from *L. ramosa*
t_½_: enzyme half-life
CMC: carboxymethylcellulose
CMCase: carboxymethylcellulase
SCB: sugarcane bagasse
PDA: potato dextrose agar
MFE: minimum free energy
SDS–PAGE: sodium dodecyl sulfate polyacrylamide gel electrophoresis
MW: molecular weight
RBB-X: Remazol Brilliant Blue linked to xylan
TLC: thin layer chromatography
X: xylanase *Lr*XynA from *L. ramosa*
A: cellulase from *A. niger*
T: cellulase from *T. viridae*
SEM: scanning electron microscopy
